# About Unsuccessful Responders to Diet and Physical Activity Interventions: A Focus on Energy Balance and Body-Weight Loss

**DOI:** 10.3390/nu18020195

**Published:** 2026-01-07

**Authors:** Angelo Tremblay, Raphaëlle Jacob, Louis Pérusse, Vicky Drapeau

**Affiliations:** 1 Department of Kinesiology, Université Laval, Québec, QC G1V 0A6, Canada; louis.perusse@kin.ulaval.ca (L.P.); vicky.drapeau@kin.ulaval.ca (V.D.); 2 Centre Nutrition, Santé et Société (NUTRISS), Institut sur la Nutrition et les Aliments Fonctionnels (INAF), Université Laval, Québec, QC G1V 0A6, Canada; 3 Quebec Heart and Lung Institute Research Center, Université Laval, Québec, QC G1V 0A6, Canada; 4 Department of Family Relations and Applied Nutrition, University of Guelph, Guelph, ON N1H 2W1, Canada; raphaell@uoguelph.ca

**Keywords:** obesity, body fat, appetite, thermogenesis

## Abstract

It is difficult to imagine that an individual living with obesity may gain body weight in response to a diet and physical activity program aiming at a negative energy balance. However, this type of case is a matter of usual occurrence in obesity clinics and has been traditionally explained by a lack of adherence to guidelines. While a link between adherence to a weight loss intervention and its outcome has been demonstrated, there is growing evidence showing that unsuccessful response to weight-reducing programs may happen in some individuals despite adequate adherence, be it imposed experimentally or spontaneously expressed in a free-living context. As described in this paper, the response to a weight loss program may range from a weight gain to a greater than expected weight loss. Based on our research findings and available literature, an unsuccessful body-weight response is seen in 5% to 20% of individuals and is attributable to behavioral and metabolic changes affecting appetite control and thermogenesis. Experimental evidence also shows that the response to a negative-energy balance is genetically determined. Globally, these observations emphasize the importance of future research in precision medicine to develop treatment approaches that progressively become more individualized.

## 1. Introduction

Obesity is the outcome of the influence of numerous environmental factors and genetic variants that promote a positive energy balance via multiple mechanisms. Its multifactorial nature has been largely investigated and has been a matter of special attention in the context of weight-reducing interventions. Not surprisingly, weight loss programs reveal extreme responses, ranging from a full success reflected by a weight loss corresponding to the theoretical energy deficit to an absence of weight loss or even weight gain. How can we explain the failure to succeed in non-responders or adverse responders under conditions where everything might have been adequately planned to induce weight loss? Traditionally, the answer would have been a lack of adherence and/or an intentional deviation from the prescribed intervention. As described in this paper, the answer is more complex than simply referring to a potential lack of integrity to explain the inability of some individuals to achieve body-weight loss under conditions of supervised energy deficit. To document this issue, this article addresses specific questions to better understand the difficulties encountered by unsuccessful responders and to propose a global vision of what may be an adequate intervention to optimize treatment outcome. Unsuccessful responders are individuals who do not achieve the expected weight reduction despite adequate adherence, while adverse responders show paradoxical weight gain or unfavorable metabolic changes under similar conditions. For clarity, the term unsuccessful responders will be mainly used throughout this paper.

## 2. What Is the Potential Magnitude of Individual Differences in Weight Loss Under Well-Controlled Living Conditions?

The deviation in energy balance from a targeted value can occur via voluntary changes in diet, physical activity, and sleep habits. It can also result from unconscious metabolic changes affecting the control of energy intake and expenditure. Finally, it is inevitably partly attributable to errors of estimation and assumptions related to the calculation of a theoretical energy deficit. Globally, this complicates the determination of what may cause a deviation, which generally cannot be clearly explained when adequate experimental controls are not performed.

This type of investigation was performed in monozygotic twins who were subjected to a protocol aiming at the characterization of human variations in energy balance under strictly controlled environmental conditions [1]. The study included a first phase of testing during which energy intake for weight maintenance was established over two weeks. Mean energy intake, adjusted for the energy equivalent of morphological changes, was used to determine each participant’s daily individual energy intake over a subsequent 100-day intervention. During this intervention, supervised physical activity was performed almost every day (93 out of 100 days) for a duration that induced a daily energy deficit of 1000 kcal/day. In addition to the experimental controls over daily energy intake and the exercise prescription, participants were required to remain sedentary and stay in the metabolic ward or its immediate surroundings. Despite this strictly controlled environment, body-weight loss ranged between 1 and 8 kg. As further discussed, the twin resemblance suggested that genetics may contribute to the intervention [1].

Even if the focus here is on body-weight loss, it is worth noting that a similar study was conducted by the same research team to examine the response to experimental overfeeding in monozygotic twins. As previously reported [2], large individual differences in body weight and fat gain were observed in response to an energy surplus of 1000 kcal/day. Significant intrapair twin resemblance in morphological changes was also found in this study.

In summary, there is evidence suggesting that even under strictly controlled laboratory conditions that practically prevent any lack of adherence, a large interindividual deviation in energy balance may be observed.

## 3. What Is the Proportion of Unsuccessful Responders to Diet and Physical Activity?

The documented relationship between adherence and weight loss in response to various interventions [3,4,5] still complicates the answer to this question. In fact, the key question here is the following: what is the proportion of unsuccessful responders to a given intervention independently of the adherence to related guidelines? Ideally, this issue should be investigated under conditions where non-adherence to guidelines is not possible. This was performed in the HERITAGE Study [6] in which participants were subjected to a 20-week exercise-training program under highly standardized conditions. Specifically, the intensity of exercise was initially fixed at a heart rate associated with 55% baseline VO_2_max for 30 min per session and was gradually increased to 75% VO_2_max for 50 min per session at Week 14. Both exercise intensity and duration were maintained at this level for the remaining six weeks. Non-adherence was not possible in this context since the ergocycle exercise prescription was individually determined and closely supervised by an exercise specialist. Despite this careful monitoring, 8% to 13% of participants displayed counterintuitive changes in cardiometabolic biomarkers, i.e., an adverse change greater than the technical measurement error for systolic blood pressure and circulating levels of insulin, triglycerides, and HDL-cholesterol. The results also showed that about 7% of participants experienced a deterioration in two or more cardiometabolic risk factors. The HERITAGE study also identified adverse responders in VO_2_max [7].

The research experience of the HERITAGE Study has pioneered research in the field of nutrition to determine if “real” unsuccessful responders to a low-energy diet (LED) exist under well-standardized dietary supervision. In the PREVIEW study [8,9], a three-year diet–exercise protocol was initiated by a 2-month supervised LED intervention, in response to which subjects had to lose at least 8% of baseline body weight to be admitted to the rest of the study. Among the total study sample of 1352 females and 668 males, the proportion of unsuccessful responders was 10.2% and 7.9%, respectively [10]. These results are concordant with those of a preliminary nutritional study, which showed that among a group of 75 women living with obesity submitted to a restricted diet, 11 participants gained body weight during the intervention [11]. This also agrees with another study that examined the issue of unsuccessful responders to different supervised diets [4]. Based on individual-reported data, body-weight gain was observed in about 9% of participants subjected to an Optifast program, whereas this proportion exceeded 21% in those submitted to a food-based program. Additional relevant information was collected in the CHANGE Project (Canadian Health Advanced by Nutrition and Graded Exercise), a one-year primary care lifestyle intervention combining individualized dietary counseling and supervised physical activity for adults with metabolic syndrome. In this study, 12 out of 101 participants were identified as adverse responders to diet and exercise for both systolic and diastolic blood pressure despite their good adherence to the program [12].

Taken together, these observations show that a minority of individuals seem to be unable to achieve the intended benefits of well-designed diet and/or physical activity interventions. Interestingly, this phenomenon is observed even under conditions where participant adherence was closely monitored and less likely to account for the observed variability in outcomes.

## 4. What Is the Profile of Unsuccessful Responders to Diet and Physical Activity?

According to the popular belief pertaining to a lack of rigor and discipline in unsuccessful responders, there is evidence linking adherence to a weight loss program and the outcome of the intervention. For instance, in the context of a diet-based weight loss program, reduced adherence was associated with reduced weight loss [5]. On the other hand, as discussed above, an adverse response to a well-standardized intervention can also be observed even if the personal willingness of participants to comply cannot influence the study outcome. As discussed in this section, the ability to respond to an intervention theoretically beneficial to everybody is a complex issue that depends on genetic and environmental influences.

As indicated above, about 10% of participants tested in the HERITAGE Study displayed an adverse response to exercise training for one cardiometabolic risk factor. This adverse response to standardized exercise was cross-validated in five other large studies and appeared independent of the use of medications [6].

The study of the profile of low responders to diet benefited from the design of the PREVIEW Study [10]. As described above, each participant followed a two-month LED regimen to lose at least 8% of baseline body weight, which was the criterion to be eligible to pursue the three-year intervention. In this context of severe energy restriction, where body weight gain was practically impossible, subjects were classified as unsuccessful and successful responders for weight loss. The adherence to diet, as measured by attendance at the bi-monthly supervision meetings, was comparable in the two groups. At baseline, body weight was slightly higher in unsuccessful responders, and they also displayed greater levels of perceived stress, resting heart rate, and dietary restraint. Interestingly, changes in these stress-related variables were also associated with the change in the main outcome of the LED intervention, i.e., percent body-weight loss. This observation is concordant with the demonstration that adrenalectomy prevents obesity [13,14]. It is also in agreement with genetic studies showing that the polymorphism in the glucocorticoid receptor gene is related to fat deposition [15] and long-term body-weight/fat gain [16].

The PREVIEW Study also provided evidence that LED may accentuate the vulnerability of unsuccessful body-weight responders by favoring less beneficial or detrimental changes in hunger, satiety, and sweet taste [10]. Finally, this study revealed that unsuccessful responders benefited to a lesser extent from some favorable effects of the program, including those related to sleep duration and quality.

The experience of our research team in the study of variations in diet-based weight loss interventions also allowed us to characterize participants who respond in the opposite of what is expected from nutritional supervision. At baseline, we found that variations in daily energy intake and resting energy expenditure did not permit the prediction of the body-weight response to the diet supervision. However, even if nutrition counseling and dietary guidelines were similar for all participants, the decrease in daily energy intake was less pronounced in unsuccessful responders (lower tertile), who also displayed a lower decrease in susceptibility to hunger compared to successful responders (upper tertile) [11]. In agreement with the PREVIEW experience, our results showed that those who gained weight while dieting exhibited a lower improvement in susceptibility to hunger. Contrary to successful responders, they were unable to benefit from the intervention to improve their sleep duration and quality [10], which are known to affect the control of appetite [17] and body composition [18,19].

In a recent study, Ard et al. [4] examined the profile of unsuccessful responders, including some weight gainers, in response to different diets. The responder status was significantly related to ethnicity and type 2 diabetes. Furthermore, the unsuccessful responders to the food-based approach also reported a greater number of previous weight loss attempts, and their Eat-26 score, which reflects symptoms and concerns characteristic of eating disorders, was higher than that of successful responders.

It is likely that the ability to respond to diet and exercise is partly influenced by the genetic profile. This agrees with the observation that, in the HERITAGE Project, the response pattern to exercise training was more homogenous within families [6]. This is also concordant with intervention studies performed in monozygotic twins, which showed a significant gene x diet or exercise interaction effect on body fat and related variables [1,2]. Beyond the study of gene x treatment interaction effects, research in genetics has recently considered mediation analyses in the study of gene-environment interaction in obesity. These analyses showed that eating behaviors [20], poor diet quality and intake of specific food groups [21], as well as sodium and aerobic fitness [22] mediate the association between a polygenic risk score (PRS) of obesity and BMI. With respect to the study of unsuccessful responders, mediation analyses could eventually be used to identify mediators of the relationship between a polygenic risk score (PRS) of obesity and weight loss and metabolic outcomes in response to various diet and exercise interventions, which would allow for improving the characterization of their profile and relevant intervention approaches.

## 5. Does the Case of Unsuccessful Responders Reflect an Incomplete Understanding of Obesity Determinants?

The characterization of the vulnerability of some individuals cannot be adequately performed without a complete understanding of what may cause a problem. In this regard, obesity research pursues the demonstration of the multifactorial nature of the problem by documenting the impact of determinants and mechanisms that were not suspected some years ago. This is the case for the recent demonstration of a link between excess sodium intake and the risk of being overweight, independently of the effect of fat and sugar intake [22]. Interestingly, an interaction effect between sodium intake and aerobic fitness was also observed in women.

Research pertaining to nutrition in relation to environmental living conditions has also revealed intriguing findings. For instance, Hersoug et al. [23] reported relevant literature and preliminary data suggesting the existence of an association between inspired CO_2_ and energy balance. According to this hypothesis, an increase in inspired CO_2_ could induce a small decrease in the pH of body fluids even if the acid-base balance is regulated. This small change in pH would be detected by the orexin system, which would initiate changes in appetite control, leading to a positive energy balance. Surprisingly, to our knowledge, the testing of this hypothesis has not been pursued, even if it is central in the current global crisis of greenhouse gas emissions and climate warning.

From a mechanistic standpoint, the discovery of a greater than previously believed involvement of the microbiota in the risk of obesity likely represents the main research development over the last two decades. The gut microbiota is influenced by diet composition, such as a high-fat diet and high-fiber intake [24,25,26], and its profile differs between individuals with obesity and those of normal weight [27]. These observations have prompted studies examining the effects of probiotic supplementation, which has been shown to favor body fat loss [28,29]. Furthermore, the observation that probiotic supplementation is associated with beneficial effects on food and mood-related behaviors in obesity [30,31] raises the perspective of an involvement of the gut microbiota via a gut–brain axis. Finally, with respect to the genetics of obesity, it is not yet known if there is an influence of the genome of the microbiota on the genetic risk score of people.

These examples tend to show that despite the important progress that has been recently achieved in the study of obesity, a lot of work has still to be performed to identify the complexity of factors related to obesity and the mechanisms by which they operate. In the meantime, the condition of unsuccessful responders to weight loss will partly remain a matter of uncertainty and hesitation.

## 6. Can Successful Responders Become Unsuccessful Maintainers?

Clinical and research experience clearly show that substantial weight loss is generally followed by weight regain over time. Even if this phenomenon has been largely documented [32], it is not yet possible to establish if the “yo-yo” cycle results in a metabolic imprint that impairs subsequent body weight maintenance. In fact, this knowledge gap is not surprising since testing the impact of a weight loss–weight regain cycle from the perspective of a persisting metabolic handicap is necessarily a matter of ethical limitation. In this regard, our team was fortunate to study explorers who imposed an extreme weight cycling experience in cross-country skiing expeditions through Greenland and Antarctica [33,34]. Specifically, we performed whole-body indirect calorimetry measurements before the expedition, and again after the expedition, when explorers had fully recovered their pre-expedition body weight and composition. Both measurements were performed under the same nutritional and physical activity conditions. As expected, the results showed that despite the strict standardization of measurement conditions, the weight cycle imposed by the expedition induced a decrease in daily energy expenditure ranging between 1.0 and 1.5 MJ.

**Figure 1:**
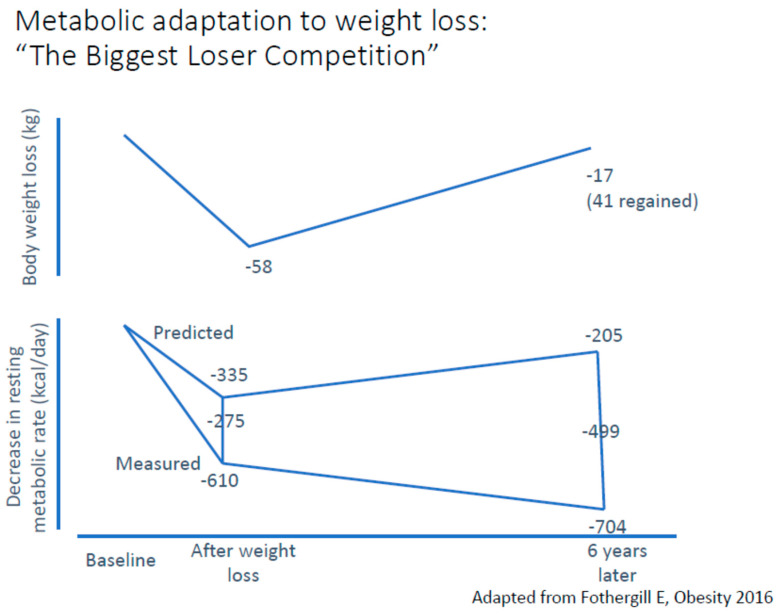
Changes in predicted and measured resting metabolic rate according to variations in body weight in the «The Biggest Loser Competition». Adapted from Fothergill et al. [35].

Subsequently, the team of Professor Kevin Hall had the opportunity to test participants in the Biggest Loser Competition [35]. As shown in Figure 1, participants were tested at baseline, following the competition after having reached a mean 58 kg weight loss, and six years later, when a mean 41 kg weight regain was observed. This figure also shows that the competition reduced resting metabolic rate (RMR) by 610 kcal/day, which exceeded by 275 kcal/day the change predicted by morphological changes. Surprisingly, the weight regain that occurred after the competition was not accompanied by an increase in RMR. On the opposite, RMR slightly decreased over the 6-year follow-up, and the gap between predicted and measured RMR, also named “adaptive thermogenesis”, was substantially increased. The results also revealed that this thermogenic adaptation was associated with reduced circulating levels of leptin and thyroid hormones.

The ability to maintain body-weight loss was also investigated in the RESOLVE Study, which was initiated by a 3-week in-house intervention combining diet restriction and a demanding physical activity regimen [36]. This first phase of the program was followed by a 1-year monitoring period during which participants were encouraged to maintain the initial diet–activity guidelines. As expected, large variations in body-weight loss maintenance were observed among the 100 participants of this study. Of particular interest was the comparison of the 13 subjects who regained more than their initial weight loss (regainers) and the 46 participants who accentuated their weight loss (losers) during the follow-up. As previously reported [36], the total adherence score to diet and activity was lower in those who regained weight than in those who maintained weight loss. Beyond this difference, those losing weight benefited from a hormonal gradient facilitating their appetite control. Specifically, plasma ghrelin, an orexigenic hormone, increased at three and six months and then unexpectedly decreased during the second half of the follow-up, even if body weight continued to decrease. This response was blunted in those who regained weight, providing additional support to the idea that successful and unsuccessful responders are distinct regarding their ability to benefit from weight loss to favorably influence appetite control and energy balance.

In a recent comprehensive review of the literature, van Baak and Mariman [37] analyzed the involvement of fat cells in the adaptive response to body-weight changes. They reported evidence showing that weight loss induced variations in cellular stress, extracellular matrix modeling, inflammatory responses, adipokine secretions, and lipolysis. However, they have not documented the impact of body fat loss on blood and tissue concentrations of organic pollutants, although it is known that an increase in these chemicals may contribute to systemic inflammation and adipose tissue dysfunction [38,39]. In this regard, our experience revealed that the increase in pollutant levels induced by weight loss is also related to a decrease in energy expenditure [40,41], thyroid hormones [40] and skeletal muscle oxidative capacity [42].

In summary, these observations suggest that exposure to a prolonged severe negative energy balance can leave metabolic imprints that might subsequently complicate body-weight stability. However, it is not established whether these effects represent natural adaptations to a severe restriction or if they are mediated by an environmental interference caused by the fat-loss-induced increase in the levels of some chemicals.

## 7. What Should Be the Obesity Management of Unsuccessful Responders to Diet and Physical Activity?

Since obesity is a multifactorial problem resulting from some genetically determined vulnerability and numerous environmental influences, it is essential for its management to be based on the most individualized approach possible. However, such a discrimination capacity is not foreseeable in a proximal future, which obviously imposes more caution on health professionals in the use of traditional approaches based on diet and physical activity guidelines.

The experience of the Quebec Family Study has shown that behaviors such as eating disinhibition, or uncontrolled eating, are more predictive of variations in body weight, cross-sectionally and over time, compared to diet composition and physical activity participation [43]. This agrees with the observation that disinhibition exerts a significant mediation role between a polygenic risk score of obesity and BMI [20]. This also highlights the importance of paying adequate attention to food- and mood-related behaviors in the management of obesity.

The classical study by Foster et al. [44] documented the important gap between patient expectations about body weight and what can be achieved in the context of a successful clinical trial. For the unsuccessful responder to diet- and/or activity-based weight loss, these results have a particular resonance since they should be used as a reference to moderate expectations about the outcome of an intervention, especially body-weight loss. In practical terms, this does not mean that recommendations focusing on “healthy eating/moving/sleeping” are not relevant for unsuccessful responders, but they represent a subgroup of users who deserve close monitoring of lifestyle modalities. Beyond this approach, it will remain relevant to consider cultural, social, and economic issues of people living with obesity to optimize the outcome of weight-loss interventions.

## 8. Conclusions

**Figure 2:**
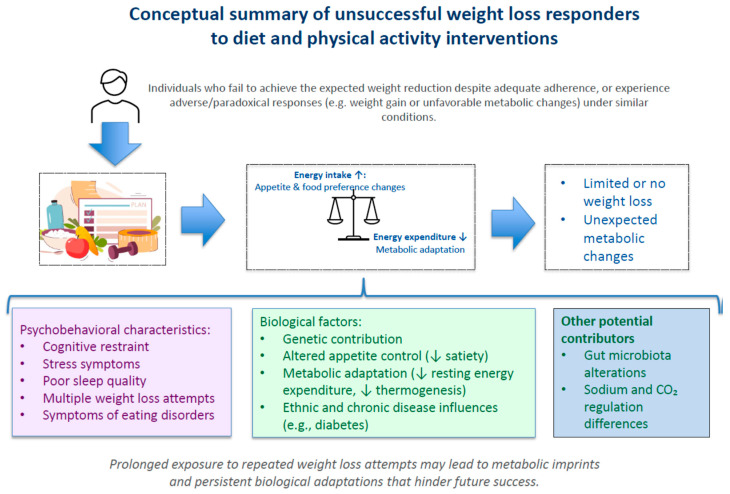
Factors potentially explaining the profile of unsuccessful weight loss responders.

The observations presented in this paper suggest that there are unsuccessful and even adverse responders to interventions that are supposedly beneficial to everybody, regardless of the willingness of these people. These adverse adaptations have been mainly documented during the course of demanding interventions, and they seem to leave long-term traces that can influence body-weight stability. As illustrated in Figure 2, there are numerous potential determinants of an unsuccessful response to a weight-loss intervention. They have not been largely studied and should be the object of subsequent research to allow precision medicine to better contribute to the management of this condition. For instance, some of these determinants highlight practical dimensions, e.g., behavioral phenotypes, stress profile, or sleep habits, that could guide individualized interventions. In the meantime, health professionals should be empathetic and perform careful follow-ups with relevant prescription adjustments in the supervision of apparently non-responders to their guidelines.

## Data Availability

No new data were created or analyzed in this study. Data sharing is not applicable to this article.

## References

[1] Bouchard C., Tremblay A., Despres J.P., Theriault G., Nadeau A., Lupien P.J., Moorjani S., Prudhomme D., Fournier G. (1994). The response to exercise with constant energy intake in identical twins. Obes. Res..

[2] Bouchard C., Tremblay A., Despres J.P., Nadeau A., Lupien P.J., Theriault G., Dussault J., Moorjani S., Pinault S., Fournier G. (1990). The response to long-term overfeeding in identical twins. N. Engl. J. Med..

[3] Alhassan S., Kim S., Bersamin A., King A.C., Gardner C.D. (2008). Dietary adherence and weight loss success among overweight women: Results from the A TO Z weight loss study. Int. J. Obes..

[4] Ard J.D., Lewis K.H., Cohen S.S., Rothberg A.E., Coburn S.L., Loper J., Matarese L., Pories W.J., Periman S. (2020). Differences in treatment response to a total diet replacement intervention versus a food-based intervention: A secondary analysis of the OPTIWIN trial. Obes. Sci. Pract..

[5] Sacks F.M., Bray G.A., Carey V.J., Smith S.R., Ryan D.H., Anton S.D., McManus K., Champagne C.M., Bishop L.M., Laranjo N. (2009). Comparison of weight-loss diets with different compositions of fat, protein, and carbohydrates. N. Engl. J. Med..

[6] Bouchard C., Blair S.N., Church T.S., Earnest C.P., Hagberg J.M., Hakkinen K., Jenkins N.T., Karavirta L., Kraus W.E., Leon A.S. (2012). Adverse metabolic response to regular exercise: Is it a rare or common occurrence?. PLoS ONE.

[7] Sarzynski M.A., Rice T.K., Despres J.P., Perusse L., Tremblay A., Stanforth P.R., Tchernof A., Barber J.L., Falciani F., Clish C. (2022). The HERITAGE Family Study: A Review of the Effects of Exercise Training on Cardiometabolic Health, with Insights into Molecular Transducers. Med. Sci. Sports Exerc..

[8] Fogelholm M., Larsen T.M., Westerterp-Plantenga M., Macdonald I., Martinez J.A., Boyadjieva N., Poppitt S., Schlicht W., Stratton G., Sundvall J. (2017). PREVIEW: Prevention of Diabetes through Lifestyle Intervention and Population Studies in Europe and around the World. Design, Methods, and Baseline Participant Description of an Adult Cohort Enrolled into a Three-Year Randomised Clinical Trial. Nutrients.

[9] Raben A., Vestentoft P.S., Brand-Miller J., Jalo E., Drummen M., Simpson L., Martinez J.A., Handjieva-Darlenska T., Stratton G., Huttunen-Lenz M. (2021). The PREVIEW intervention study: Results from a 3-year randomized 2 x 2 factorial multinational trial investigating the role of protein, glycaemic index and physical activity for prevention of type 2 diabetes. Diabetes Obes. Metab..

[10] Tremblay A., Fogelholm M., Jalo E., Westerterp-Plantenga M.S., Adam T.C., Huttunen-Lenz M., Stratton G., Lam T., Handjieva-Darlenska T., Handjiev S. (2021). What Is the Profile of Overweight Individuals Who Are Unsuccessful Responders to a Low-Energy Diet? A PREVIEW Sub-study. Front. Nutr..

[11] Tremblay A., Lepage C., Panahi S., Couture C., Drapeau V. (2015). Adaptations to a diet-based weight-reducing programme in obese women resistant to weight loss. Clin. Obes..

[12] Marin-Couture E., Filion M.J., Boukari R., Jeejeebhoy K., Dhaliwal R., Brauer P., Royall D., Mutch D.M., Klein D., Tremblay A. (2022). Relationship between Cardiometabolic Factors and the Response of Blood Pressure to a One-Year Primary Care Lifestyle Intervention in Metabolic Syndrome Patients. Metabolites.

[13] Freedman M.R., Horwitz B.A., Stern J.S. (1986). Effect of adrenalectomy and glucocorticoid replacement on development of obesity. Am. J. Physiol..

[14] Tokuyama K., Himms-Hagen J. (1989). Adrenalectomy prevents obesity in glutamate-treated mice. Am. J. Physiol..

[15] Buemann B., Vohl M.C., Chagnon M., Chagnon Y.C., Gagnon J., Perusse L., Dionne F., Despres J.P., Tremblay A., Nadeau A. (1997). Abdominal visceral fat is associated with a BclI restriction fragment length polymorphism at the glucocorticoid receptor gene locus. Obes. Res..

[16] Tremblay A., Bouchard L., Bouchard C., Despres J.P., Drapeau V., Perusse L. (2003). Long-term adiposity changes are related to a glucocorticoid receptor polymorphism in young females. J. Clin. Endocrinol. Metab..

[17] Spiegel K., Leproult R., L’Hermite-Baleriaux M., Copinschi G., Penev P.D., Van Cauter E. (2004). Leptin levels are dependent on sleep duration: Relationships with sympathovagal balance, carbohydrate regulation, cortisol, and thyrotropin. J. Clin. Endocrinol. Metab..

[18] Chaput J.P., Despres J.P., Bouchard C., Tremblay A. (2007). Short sleep duration is associated with reduced leptin levels and increased adiposity: Results from the Quebec family study. Obesity.

[19] Chaput J.P., Doucet E., Tremblay A. (2012). Obesity: A disease or a biological adaptation? An update. Obes. Rev..

[20] Jacob R., Drapeau V., Tremblay A., Provencher V., Bouchard C., Perusse L. (2018). The role of eating behavior traits in mediating genetic susceptibility to obesity. Am. J. Clin. Nutr..

[21] Jacob R., Bertrand C., Llewellyn C., Couture C., Labonte M.E., Tremblay A., Bouchard C., Drapeau V., Perusse L. (2022). Dietary Mediators of the Genetic Susceptibility to Obesity-Results from the Quebec Family Study. J. Nutr..

[22] Tremblay A., Perusse L., Bertrand C., Jacob R., Couture C., Drapeau V. (2023). Effects of sodium intake and cardiorespiratory fitness on body composition and genetic susceptibility to obesity: Results from the Quebec Family Study. Br. J. Nutr..

[23] Hersoug L.G., Sjodin A., Astrup A. (2012). A proposed potential role for increasing atmospheric CO_2_ as a promoter of weight gain and obesity. Nutr. Diabetes.

[24] Heldebrant D.J., Yonker C.R., Jessop P.G., Phan L. (2009). Reversible uptake of COS, CS_2_, and SO_2_: Ionic liquids with O-alkylxanthate, O-alkylthiocarbonyl, and O-alkylsulfite anions. Chemistry.

[25] Jumpertz R., Le D.S., Turnbaugh P.J., Trinidad C., Bogardus C., Gordon J.I., Krakoff J. (2011). Energy-balance studies reveal associations between gut microbes, caloric load, and nutrient absorption in humans. Am. J. Clin. Nutr..

[26] Wu G.D., Chen J., Hoffmann C., Bittinger K., Chen Y.Y., Keilbaugh S.A., Bewtra M., Knights D., Walters W.A., Knight R. (2011). Linking long-term dietary patterns with gut microbial enterotypes. Science.

[27] Ley R.E., Backhed F., Turnbaugh P., Lozupone C.A., Knight R.D., Gordon J.I. (2005). Obesity alters gut microbial ecology. Proc. Natl. Acad. Sci. USA.

[28] Kadooka Y., Sato M., Imaizumi K., Ogawa A., Ikuyama K., Akai Y., Okano M., Kagoshima M., Tsuchida T. (2010). Regulation of abdominal adiposity by probiotics (Lactobacillus gasseri SBT2055) in adults with obese tendencies in a randomized controlled trial. Eur. J. Clin. Nutr..

[29] Sanchez M., Darimont C., Drapeau V., Emady-Azar S., Lepage M., Rezzonico E., Ngom-Bru C., Berger B., Philippe L., Ammon-Zuffrey C. (2014). Effect of Lactobacillus rhamnosus CGMCC1.3724 supplementation on weight loss and maintenance in obese men and women. Br. J. Nutr..

[30] Choi B.S., Brunelle L., Pilon G., Cautela B.G., Tompkins T.A., Drapeau V., Marette A., Tremblay A. (2023). Lacticaseibacillus rhamnosus HA-114 improves eating behaviors and mood-related factors in adults with overweight during weight loss: A randomized controlled trial. Nutr. Neurosci..

[31] Sanchez M., Darimont C., Panahi S., Drapeau V., Marette A., Taylor V.H., Dore J., Tremblay A. (2017). Effects of a Diet-Based Weight-Reducing Program with Probiotic Supplementation on Satiety Efficiency, Eating Behaviour Traits, and Psychosocial Behaviours in Obese Individuals. Nutrients.

[32] Anderson J.W., Konz E.C., Frederich R.C., Wood C.L. (2001). Long-term weight-loss maintenance: A meta-analysis of US studies. Am. J. Clin. Nutr..

[33] St-Pierre S., Roy B., Tremblay A. (1996). A case study on energy balance during an expedition through Greenland. Int. J. Obes. Relat. Metab. Disord..

[34] Tremblay A., Major G., Doucet E., Trayhurn P., Astrup A. (2007). Role of adaptive thermogenesis in unsuccessful weight-loss intervention. Future Lipidol..

[35] Fothergill E., Guo J., Howard L., Kerns J.C., Knuth N.D., Brychta R., Chen K.Y., Skarulis M.C., Walter M., Walter P.J. (2016). Persistent metabolic adaptation 6 years after "The Biggest Loser" competition. Obesity.

[36] Bailly M., Isacco L., Dutheil F., Courteix D., Lesourd B., Chapier R., Obert P., Walther G., Bagheri R., Vinet A. (2025). Initial and evolutionary profile of adverse responders to an intensive weight loss intervention: The RESOLVE Study. J. Sports Med. Phys. Fitness.

[37] van Baak M.A., Mariman E.C.M. (2019). Mechanisms of weight regain after weight loss—The role of adipose tissue. Nat. Rev. Endocrinol..

[38] Imbeault P., Findlay C.S., Robidoux M.A., Haman F., Blais J.M., Tremblay A., Springthorpe S., Pal S., Seabert T., Krummel E.M. (2012). Dysregulation of cytokine response in Canadian First Nations communities: Is there an association with persistent organic pollutant levels?. PLoS ONE.

[39] Rolle-Kampczyk U., Gebauer S., Haange S.B., Schubert K., Kern M., Moulla Y., Dietrich A., Schon M.R., Kloting N., von Bergen M. (2020). Accumulation of distinct persistent organic pollutants is associated with adipose tissue inflammation. Sci. Total Environ..

[40] Pelletier C., Doucet E., Imbeault P., Tremblay A. (2002). Associations between weight loss-induced changes in plasma organochlorine concentrations, serum T(3) concentration, and resting metabolic rate. Toxicol. Sci..

[41] Tremblay A., Pelletier C., Doucet E., Imbeault P. (2004). Thermogenesis and weight loss in obese individuals: A primary association with organochlorine pollution. Int. J. Obes. Relat. Metab. Disord..

[42] Imbeault P., Tremblay A., Simoneau J.A., Joanisse D.R. (2002). Weight loss-induced rise in plasma pollutant is associated with reduced skeletal muscle oxidative capacity. Am. J. Physiol. Endocrinol. Metab..

[43] Chaput J.P., Leblanc C., Perusse L., Despres J.P., Bouchard C., Tremblay A. (2009). Risk factors for adult overweight and obesity in the Quebec Family Study: Have we been barking up the wrong tree?. Obesity.

[44] Foster G.D., Wadden T.A., Vogt R.A., Brewer G. (1997). What is a reasonable weight loss? Patients’ expectations and evaluations of obesity treatment outcomes. J. Consult. Clin. Psychol..

